# Reciprocal regulatory mechanism between miR-214-3p and FGFR1 in FGFR1-amplified lung cancer

**DOI:** 10.1038/s41389-019-0151-1

**Published:** 2019-09-06

**Authors:** Ying Yang, Ziming Li, Hong Yuan, Wenxiang Ji, Kaixuan Wang, Tingting Lu, Yongfeng Yu, Qingyu Zeng, Fan Li, Weiliang Xia, Shun Lu

**Affiliations:** 1 Shanghai Lung Cancer Center, Shanghai Chest Hospital, Shanghai Jiao Tong University, West Huaihai Road 241, 20030 Shanghai, China; 2 School of Biomedical Engineering and Med-X Research Institute, Shanghai Jiao Tong University, Huashan Road 1954, 200030 Shanghai, China

**Keywords:** Lung cancer, Cancer therapy

## Abstract

MicroRNA (miRNA) and fibroblast growth factor receptor 1 (FGFR1) dysregulation are considered to play an important role in tumor proliferation, invasion, and metastasis. However, the regulatory mechanism between miRNAs and FGFR1 in lung cancer remains unclear and extremely critical. miR-214-3p was sharply decreased and showed a significantly negative correlation with FGFR1 in lung cancer patients (*n* = 30). Luciferase reporter assay confirmed that miR-214-3p could downregulate FGFR1 by directly targeting 3′-untranslated region (UTR). miR-214-3p inhibited the processes of epithelial–mesenchymal transition and Wnt/MAPK/AKT (Wnt/mitogen-activated protein kinase/AKT) signaling pathway by targeting FGFR1. Moreover, miR-214-3p not only established a negative feedback regulation loop with FGFR1 through ERK (extracellular signal-regulated kinase) but also developed a synergism with FGFR1 inhibitor AZD4547. In conclusion, our study demonstrated the regulatory mechanism between miR-214-3p and FGFR1 in lung cancer. miR-214-3p acts as a vital target in FGFR1-amplified lung cancer by forming a miR-214-3p-FGFR1-Wnt/MAPK/AKT signaling pathway network. Co-targeting miR-214-3p and FGFR1 could provide greater benefits to patients with FGFR1-amplified lung cancer.

## Introduction

Worldwide, lung cancer with a 5-year survival rate of 18% leads the cancer incidence and mortality, and accounts for almost 20% cancer deaths^1,2^. Lung cancer is mainly composed of small-cell lung cancer (SCLC 10–15%) and non-small-cell lung cancer (NSCLC, 80–85%). NSCLC is subdivided into adenocarcinoma, squamous cell carcinoma (SQCC), large cell carcinoma, and so on^3,4^. Early studies have already demonstrated that local progression and distant metastasis, possibly associated with epithelial–mesenchymal transition (EMT), are the most common obstacles in the treatment of lung cancer^5^. Targeted drugs have been developed in lung adenocarcinoma (LADC) to inhibit tumor proliferation and invasion. The discovery of molecularly targeted therapies for patients with EGFR mutation and ALK or ROS1 rearrangements has been a breakthrough in the treatment history of LADC^6^. However, only a few genomic alterations of lung squamous cell carcinoma (LSQCC) and SCLC have been discovered, and the corresponding molecularly targeted drugs have not been used for clinical treatment^7^. Fibroblast growth factor receptor (FGFR) dysfunction is fairly common in a variety of cancers^8^. Meanwhile, FGFR1 amplification is the most common type, with 20% in LSQCC, 5–7% in SCLC, and 1–3% in LADC^8–11^. AZD4547 and BGJ398 are selective inhibitors of FGFR1, 2, and 3 in phase Ib clinical trials^12–16^, whereas LY2874455 is a pan-FGFR inhibitor in phase I clinical trials^17,18^. FGFR1-amplified lung cancer cell lines have shown promising preclinical sensitivity to kinase inhibition^19–21^. However, the effect of these FGFR1 inhibitors are modest. It is known that FGFR1 can facilitate tumor development by promoting EMT in various cancers, including lung cancer, gastric cancer, prostate cancer, and breast cancer^16,22–24^. EMT is also considered to be one of the key factors for drug resistance^25^. Therefore, as a promising target in LSQCC and SLCL, there is an urgent need to further explore the development and progression of FGFR1 in FGFR1-amplified lung cancer.

MicroRNAs (miRNAs) are a class of endogenous and small non-coding RNAs (20–24 nucleotides) that regulate a large variety of cellular processes, including differentiation, apoptosis, and proliferation, as well as EMT^26–29^. Through the binding to the 3′-untranslated region (3′-UTR) of target genes, miRNAs could function as either tumor suppressors or promoters^30^. miRNAs are dysregulated in many types of tumors^31^. To date, studies have unveiled a large amount of miRNA signatures in lung cancer, yet miRNA dysregulation in FGFR1-amplified lung cancer has remained unclear. Therefore, it is necessary to explore the regulatory mechanism between miRNAs and FGFR1 in FGFR1-amplified lung cancer, which may help explain the modest suppression of lung cancer by FGFR1 inhibitors, and, more importantly, develop novel and better therapies.

## Results

### Reduced miR-214-3p expression confers poor survival

**Fig. 1:**
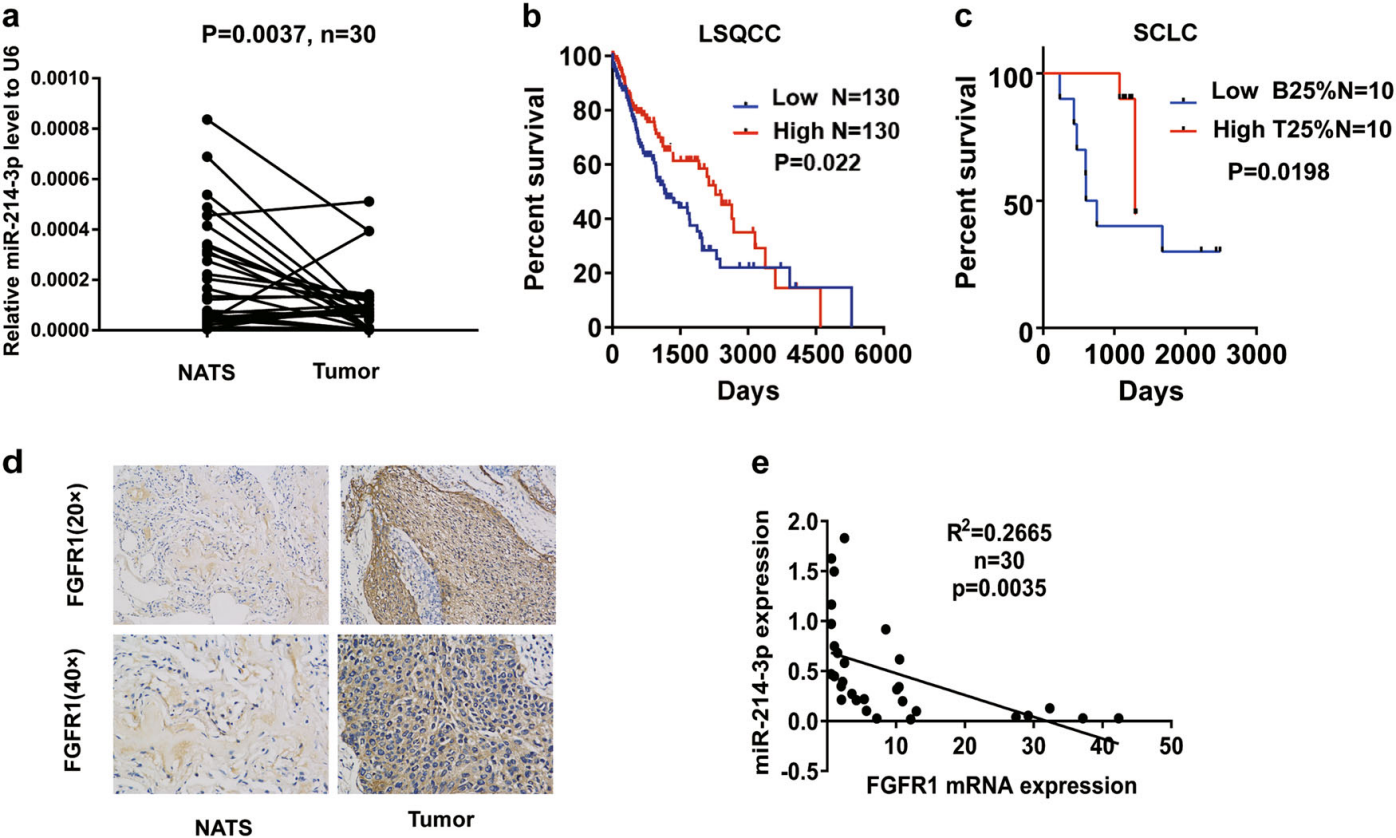
miR-214-3p expression deregulation correlated with survival rate. **a** Relative miR-214-3p expression levels was detected by quantitative real-time PCR (qRT-PCR) in lung tumor tissues and non-tumor adjacent tissues (NATs), **b** Kaplan–Meier curves depicting overall survival (OS) according to the expression of miR-214-3p in lung squamous cell carcinoma (LSQCC) patients in The Cancer Genome Atlas (TCGA) database (*n* = 260), **c** Kaplan–Meier curves depicting OS according to the top 25% and bottom 25% expression of the miR-214-3p in small-cell lung cancer (SCLC) patients in GSE27435 microRNA (miRNA) profiles, **d** the immunohistochemistry (IHC) staining of fibroblast growth factor receptor 1 (FGFR1) in lung tumor tissues and NATs, **e** linear fit correlation analysis between FGFR1 messenger RNAs (mRNA) and miR-214-3p were conducted in lung cancer patients (*n* = 30). *P* values were calculated by paired *t* test

To determine the correlation between miRNA and the progression of lung cancer, we examined 10 metastasis-associated miRNAs in 30 patients with LSQCC (Fig. S1). Quantitative real-time PCR (qRT-PCR) demonstrated a lower expression of miR-214-3p in the tumor tissues of lung cancer (*n* = 30) than in non-tumor adjacent tissues (NATs) (Fig. 1a), while immunohistochemistry (IHC) showed a higher FGFR1 level in the tumor tissues of lung cancer than in the NATs (Fig. 1d). A Kaplan–Meier analysis verified longer overall survival (OS) in LSQCC patents with high miR-214-3p in The Cancer Genome Atlas (TCGA) database (Fig. 1b). The miRNA profiles of SCLC in GSE27435 also demonstrated that longer OS in SCLC patients with high miR-214-3p (Fig. 1c). Negative correlation between miR-214-3p and FGFR1 was confirmed by qRT-PCR (Fig. 1e). The above results revealed that both miR-214-3p and FGFR1 are involved in lung cancer progression.

### miR-214-3p suppressed the proliferation, migration, and invasion of FGFR1-amplified lung cancer cells

**Fig. 2:**
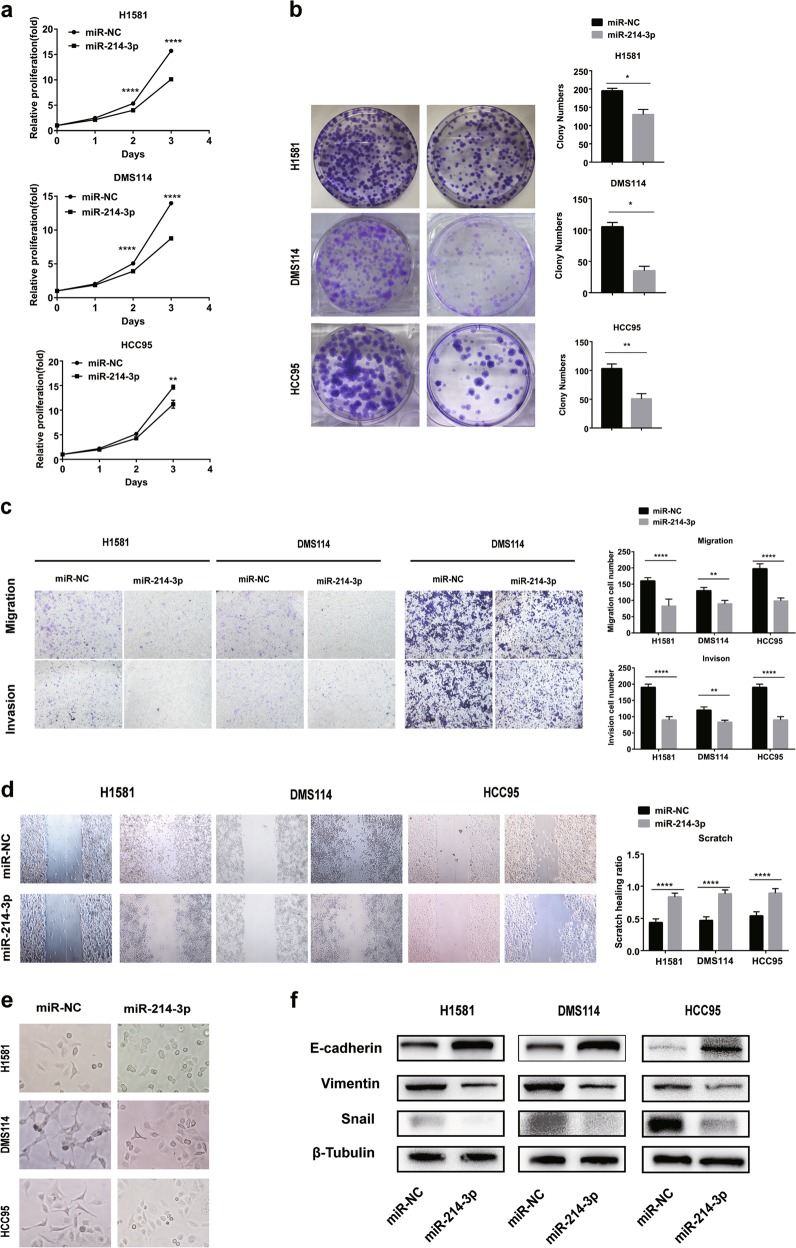
miR-214-3p suppressed the proliferation, epithelial–mesenchymal transition (EMT) process, and invasion in fibroblast growth factor receptor 1 (FGFR1)-amplified lung cancer cell lines. H1581, DMS114, and HCC95 cell lines were transfected with miR-214-3p mimic or miR-NC. **a**, **b** Cell growth was measured by the CCK8 assay and colony assay. **c**, **d** Migration and invasion was determined by scratch assay and transwell assay. **e** Representative images showing the altered morphological characteristics of the cells. **f** Quantification of EMT markers was measured by western blot. *P* values were calculated by Student’s *t* test: *p < 0.05; **p < 0.01; ****p < 0.0001

To explore the role of miR-214-3p in FGFR1-amplified lung cancer cells, H1581, DMS114, and HCC95 cells with high expression of FGFR1 were used and were authenticated by short tandem repeat (STR) profiling (Table S1)^19–21^. Both cells were transfected with miR-214-3p mimics or inhibitors. miR-214-3p inhibited proliferation in all of the cell lines, as determined by the CCK8 assay and colony formation (Fig. 2a, b). miR-214-3p inhibited migration and invasion as determined by scratch assay and transwell assay (Fig. 2c, d). The altered morphological characteristics of the cells caused by the inhibition of EMT process by miR-214-3p were presented (Fig. 2e). Moreover, miR-214-3p overexpression resulted in the downregulation of mesenchymal markers including vimentin (VIM) and Snail, as well as the upregulation of epithelial markers such as E-cadherin (E-cad) and ZO-1 in both H1581 and DMS114 cells (Fig. 2f).

Conversely, the downregulation of miR-214-3p promoted proliferation, migration, and invasion in both cell lines (Fig. S2), inhibited the levels of epithelial markers, and induced the expression of mesenchymal markers (Fig. S2).

These results indicated that miR-214-3p blocked the EMT process in FGFR1-amplified lung cancer cell lines as a tumor suppressor.

### FGFR1 is a direct target of miR-214-3p

**Fig. 3:**
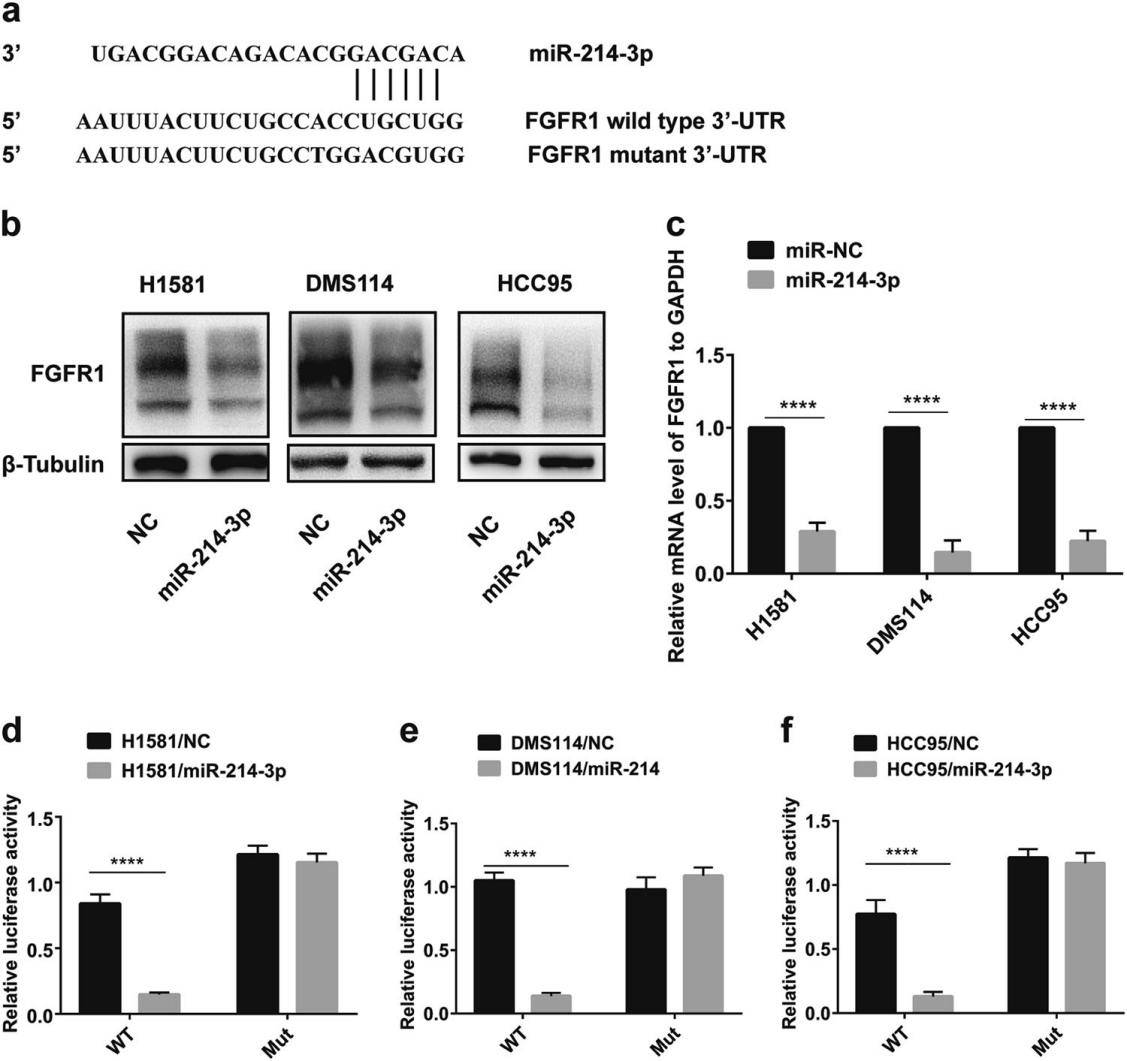
Fibroblast growth factor receptor 1 (FGFR1) is a direct target of miR-214-3p. **a** Schematic diagram of the predicted binding site of FGFR1 3′-untranslated region (UTR) and miR-214-3p. **b**, **c** Western blot and quantitative real-time PCR (qRT-PCR) analyses of FGFR1 expression levels in H1581 and DMS114 cells transfected with miR-NC or miR-214-3p. **d**–**f** Luciferase assay of the reporter plasmids including the wild-type and mutant FGFR1 3′-UTR. *P* values were calculated by Student’s *t* test. ****p < 0.0001

miRWalk 2.0 and TargetScan were used to predict the potential targets and potential binding sites of miR-214-3p, and hundreds of genes were screened out. Combined with a literature search (PubMed^©^, NCBI, Bethesda, MD, USA), we found the 3′-UTR of FGFR1 with potential miR-214-3p binding sites (Fig. 3a). Meanwhile, FGFR1 had a sharp downregulation after miR-214-3p overexpression for 48 h, which was detected by western and qRT-PCR (Fig. 3b, c). To test whether the miR-214-3p directly regulates FGFR1, mutant (FGFR1 3′-UTR-MUT) 3′-UTR, and wild type (FGFR1 3′-UTR-WT), the regions of FGFR1 gene were separately cloned into the pGL3 vector downstream of the luciferase-coding region. The luciferase reporter assay indicated that miR-214-3p reduced the luciferase activity of the WT 3′-UTR reporter in all of the cell lines, but had no effect on the MUT 3′-UTR reporter (Fig. 3d–f), which suggested that FGFR1 expression was directly regulated by miR-214-3p.

### miR-214-3p inhibits proliferation, migration, and invasion of NSCLC cells by targeting FGFR1

**Fig. 4:**
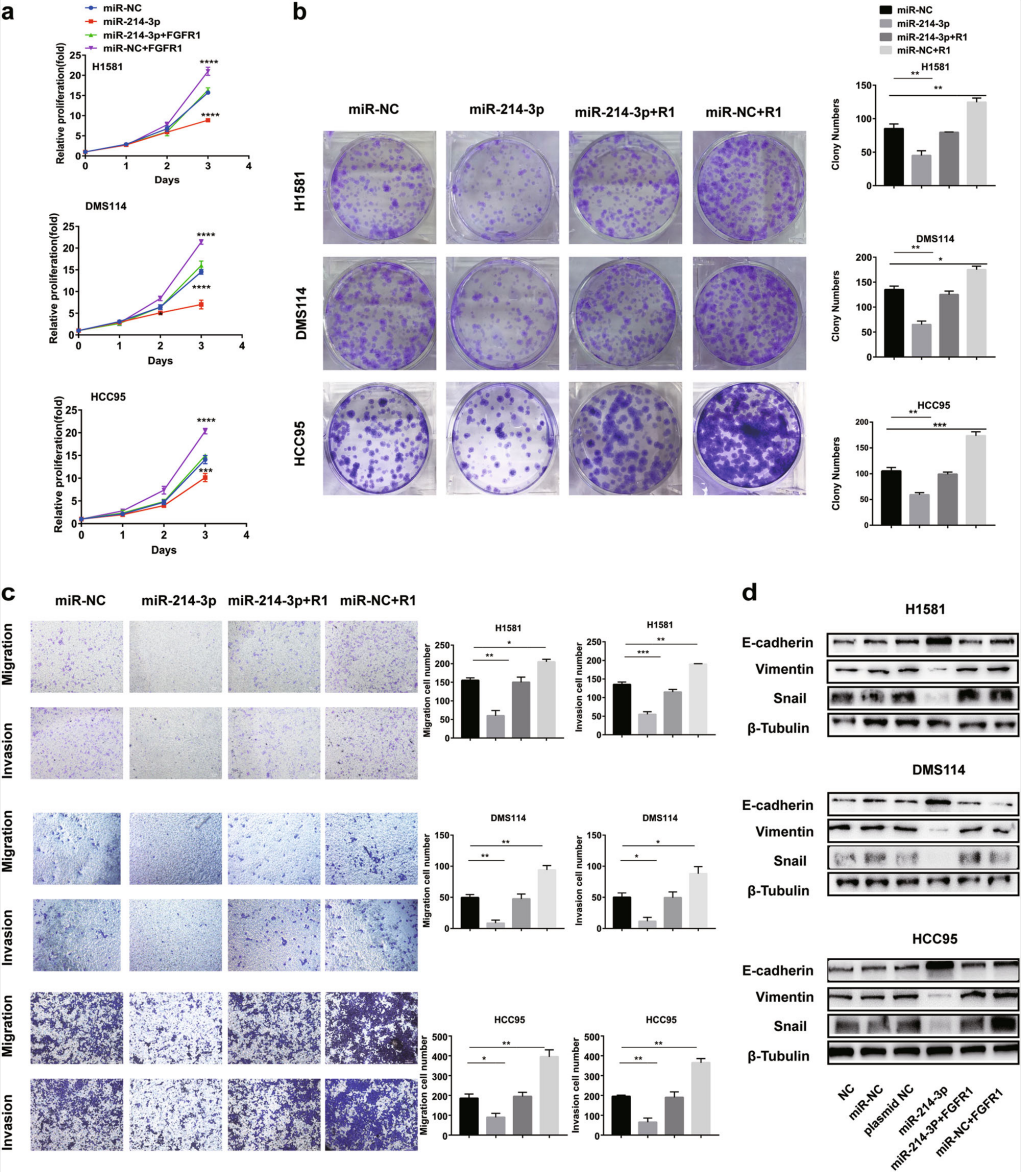
miR-214-3p inhibits proliferation, migration, and invasion of non-small-cell lung cancer (NSCLC) cells by targeting fibroblast growth factor receptor 1 (FGFR1). H1581, DMS114, and HCC95 cell lines were transfected with miR-214-3p-mimic or miR-NC or co-transfection of miR-214-3p-mimic and FGFR1 overexpression plasmid or co-transfection of miR-NC and FGFR1 overexpression plasmid. R1 in the figure represented FGFR1 overexpression plasmid. **a**, **b** Cell growth was measured by the CCK8 assay and colony assay. **c** Transwell assay was conducted to quantify the migration and invasion. **d** Quantification of epithelial–mesenchymal transition (EMT) markers was measured by western blot. *P* values were calculated by Student’s *t* test. *p < 0.05; **p < 0.01; ***p < 0.001; ****p < 0.0001

To determine whether FGFR1 acts a pivotal role in miR-214-3p-induced alterations in cell proliferation and metastasis, H1581, DMS114, and HCC95 cells transfected with FGFR1 overexpression plasmid were used. The inhibitory effects of miR-214-3p on proliferation (Fig. 4a, b) and metastasis (Fig. 4c) induced by miR-214-3p were abrogated by FGFR1 overexpression in the cells. The downregulation of mesenchymal markers, including VIM and Snail, and the upregulation of epithelial marker, E-cad induced by miR-214-3p, were also rescued by FGFR1 restoration (Fig. 4d).

### miR-214-3p suppresses oncogenic pathway by targeting FGFR1

**Fig. 5:**
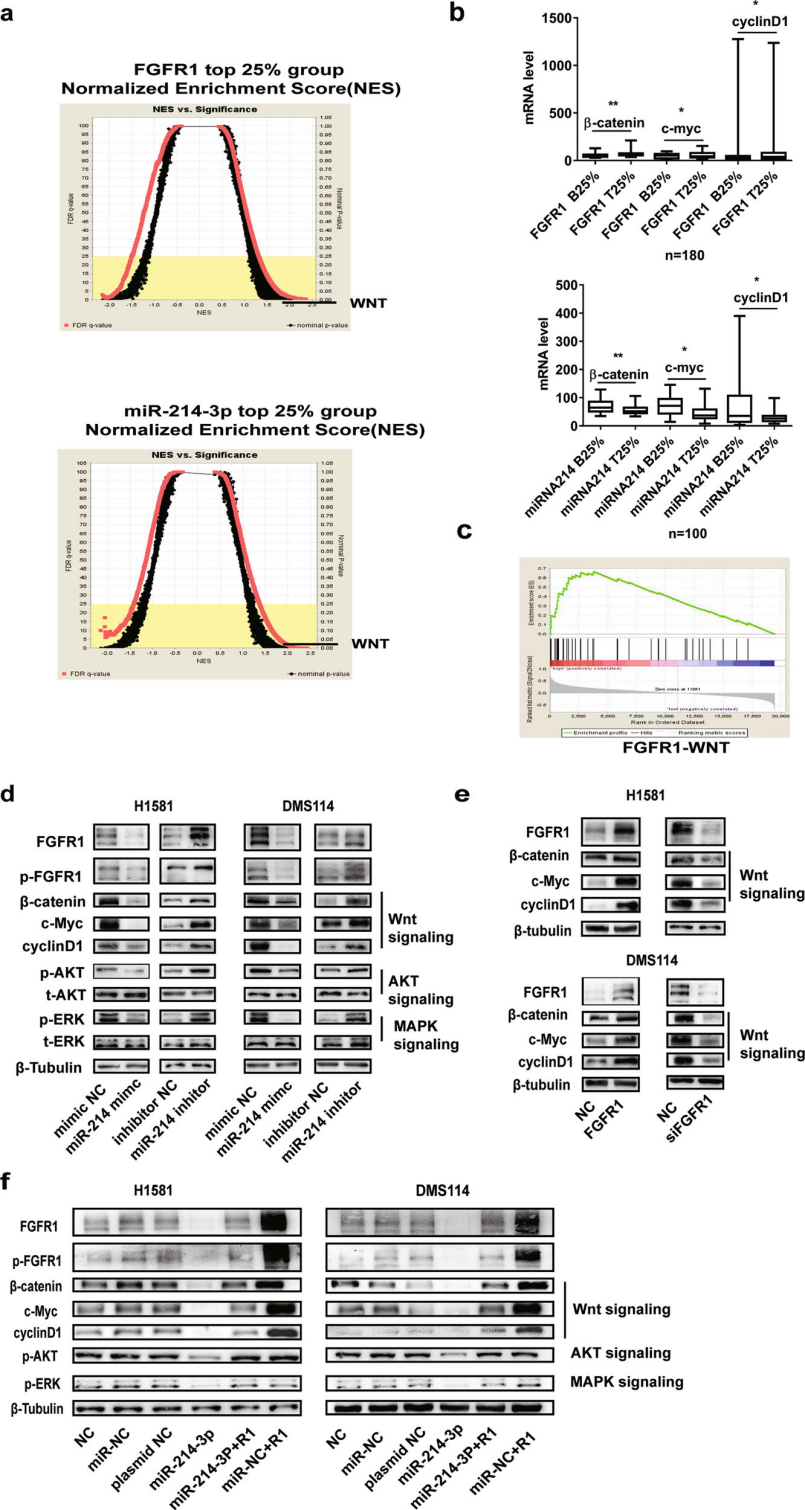
miR-214-3p suppresses oncogenic pathway by targeting fibroblast growth factor receptor 1 (FGFR1). **a**, **c** A gene set enrichment analysis related to the epithelial–mesenchymal transition (EMT) signatures and hallmarks in the groups with high and low expression of miR-214-3p or FGFR1 in the The Cancer Genome Atlas (TCGA) lung cancer cohort. The signaling pathways were negatively regulated by miR-214-3p and positively regulated by FGFR1. Wnt signaling pathway as an intersection was determined by a normalized enrichment score (NES). **b** The mRNA level of β-catenin, c-Myc, and cyclinD1 in Wnt signaling in the groups with high and low expression of miR-214-3p or FGFR1 in the TCGA lung cancer cohort. **d** After transfection of miR-NC and miR-214-3p mimic or anti-miR-NC and anti-miR-214-3p for 24 h, the protein levels of FGFR1, pFGFR1, MAPK (mitogen-activated protein kinase), phosphoinositide 3-kinase-AKT (PI3K-AKT), and Wnt signaling pathway were measured by western blot, and the expression level of FGFR1 were measured by western blot after transfection for 48 h. **e** After transfection of FGFR1 overexpression plasmid or small interfering RNA (siRNA) of FGFR1 for 48 h, the protein levels of Wnt signaling pathways were measured by western blot. **f** After transfection of miR-214-3p or FGFR1 plasmid or co-transfection of miR-214-3p and FGFR1 plasmid for 48 h, the protein levels of FGFR1, pFGFR1, MAPK, AKT, and Wnt signaling pathways were measured by western blot. *P* values were calculated by Student’s *t* test: *p < 0.05; **p < 0.01

After Gene Ontology (GO) data analysis, the pathways of FGFR1 and miR-214-3p related to EMT were explored. The Wnt signaling pathway was identified to be an intersection (Fig. 5a–c). Interestingly, MAPK/AKT (mitogen-activated protein kinase/AKT) signaling pathways as classic oncogenic pathways were regulated by both miR-214-3p and FGFR1^32–35^. Thus, the Wnt/MAPK/AKT signaling pathways were investigated in this study. Twenty-four hours after transfection of miR-214-3p-mimic, the levels of the proteins in Wnt/MAPK/AKT signaling pathways were markedly decreased (Fig. 5d). To further explore the relationship between FGFR1 and the Wnt signaling pathway, the FGFR1 overexpression plasmid and small interfering RNA (siRNA) of FGFR1 were used. FGFR1 overexpression increased the expression of β-catenin, c-Myc, and cyclinD1, while siRNA of FGFR1 decreased the expression of the above molecules (Fig. 5e). This effect of miR-214-3p on Wnt/MAPK/AKT signaling pathways was offset by FGFR1 overexpression (Fig. 5f). The major function of miRNAs is to regulate their target genes by messenger RNA (mRNA) cleavage or by inhibiting translation, which depend on the suitability with the 3′-UTR of target genes.

The results suggested that miR-214-3p could not only downregulate FGFR1 by post-transcriptional regulation but also inhibit Wnt/MAPK/AKT pathways by suppressing the phosphorylation of FGFR1.

### FGFR1 regulates the expression of miR-214-3p through ERK activation

**Fig. 6:**
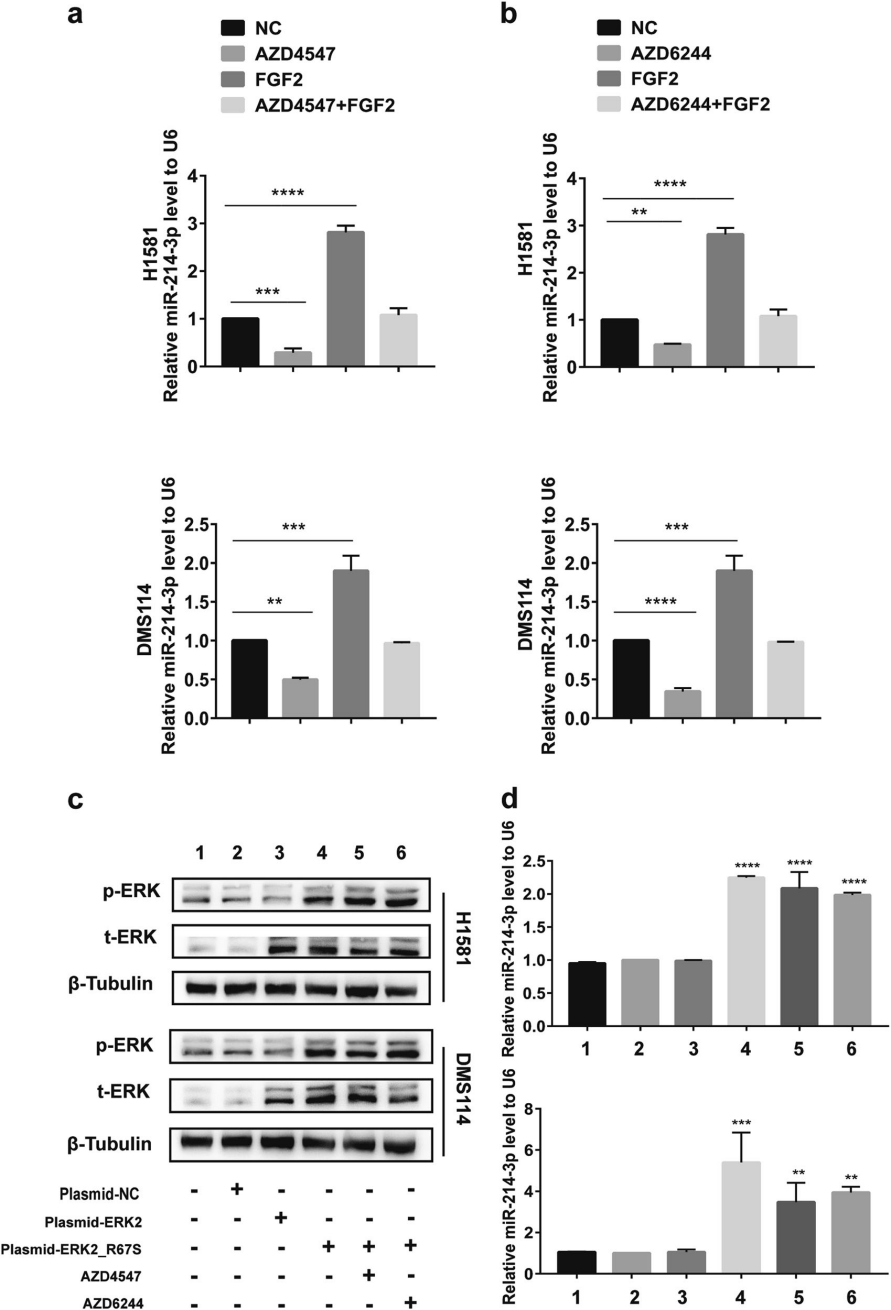
Fibroblast growth factor receptor 1 (FGFR1) regulates the expression of miR-214 by regulating extracellular signal-regulated kinase (ERK). **a** After treatment with FGF2 or FGFR1 inhibitor AZD4547 (1 μM) or FGF2 and AZD4547 in H1581 and DMS114 cell lines, the level of miR-214-3p was detected by quantitative real-time PCR (qRT-PCR). **b** After treatment with MEK/ERK inhibitor AZD6244 (1 μM) or FGF2 or FGF2 and AZD6244 (1 μM) in H1581 and DMS114 cell lines, the level of miR-214-3p was detected by qRT-PCR. **c**, **d** H1581 and DMS114 cell lines were transfected by plasmid-NC, wild-type ERK2 plasmid (plasmid-ERK2), or autophosphorylation variant ERK2_R67S plasmid (plasmid-ERK_R67S), or plasmid-ERK_R67S followed by treatment of AZD4547 or AZD6244. **c** Expression of pERK1/2 and total ERK1/2 were detected by Western blot. **d** Expression of miR-214-3p were measured by qPCR. *P* values were calculated by Student’s *t* test: **p < 0.01; ***p < 0.001; ****p < 0.0001

Interestingly, we found that miR-214-3p was downregulated by FGFR1 inhibitor AZD4547 and upregulated by the exogenous FGFR1 ligand FGF2 (Fig. 6a). To further investigate which signaling pathway downstream of FGFR1 could regulate the expression of miR-214-3p, Wnt pathway inhibitor XAV-939, the AKT pathway inhibitor MK-2206 2HCl, and the MAPK pathway inhibitor AZD6244 (Fig. S3) were used. MiR-214-3p was only suppressed by AZD6244 (Fig. 6b). AZD6244 could not suppress the level of miR-214-3p in the presence of FGF2 (Fig. 6b). H1581 and DMS114 cells were transfected with the WT extracellular signal-regulated kinase 2 (ERK2) plasmid or the dominant active ERK2_R67S plasmid. Compared with the WT ERK2 and negative control, ERK2_R67S upregulated the level of phosphorylated-ERK, which could not be suppressed by AZD4547 or AZD6244 (Fig. 6c). Meanwhile, the upregulation of miR-214-3p caused by ERK2_R67S plasmid transfection was not suppressed by FGFR1 inhibitor AZD4547 or MEK/ERK inhibitor AZD6244 (Fig. 6d). Therefore, we verified that FGFR1 regulated the expression of miR-214-3p through ERK activation.

### miR-214-3p and AZD4547 have synergistic antitumor effects in vitro and in vivo

**Fig. 7:**
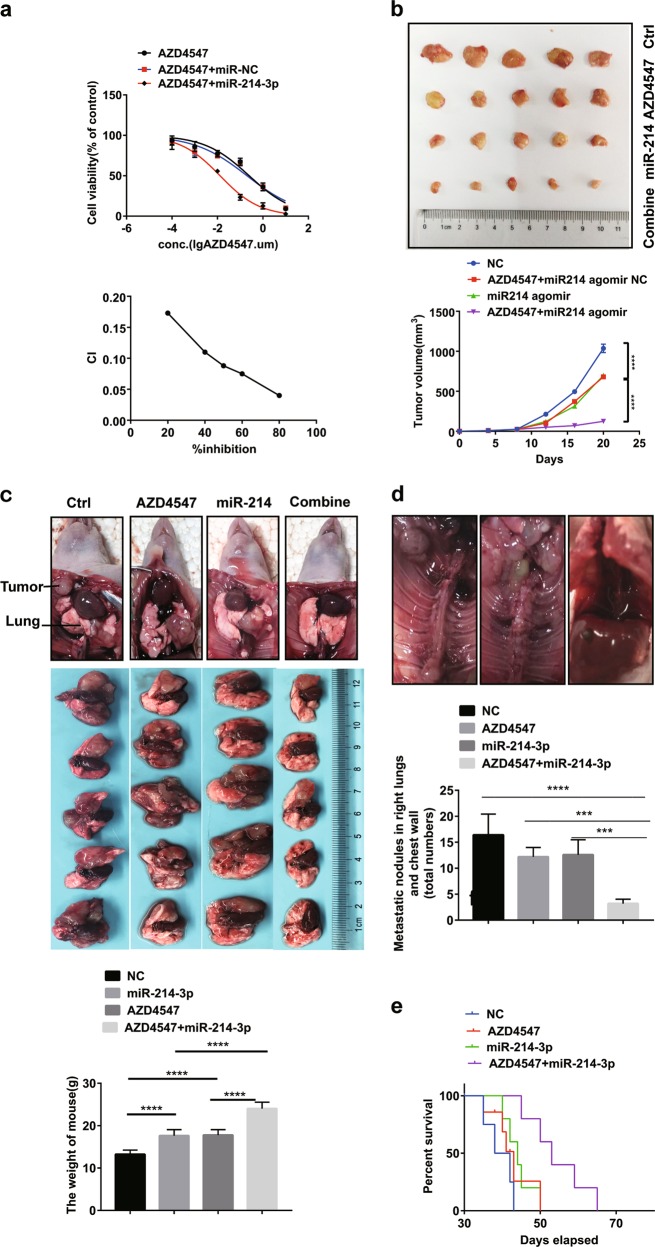
miR-214 and AZD4547 have synergistic effects in vitro and in vivo. The experiments were conduct using H1581 (this figure) and DMS114 cells (Fig. S4). **a** H1581 cells were transfected with 0.3 nM miR-NC or miR-214-3p, and then incubated with AZD4547 in a serial dilution. After 72 h, CCK8 was used to evaluate cellular proliferation. CI values were determined by non-linear regression methods at any given effect. (CI = 1, additivity; CI.1, antagonism; CI,1, synergy). **b** Representative images showing tumor formation in the nude mices treated with microRNA (miRNA) agomir NC, AZD4547, miR-214-3p agomir or miR-214-3p, and agomir for 3 weeks (combined). **c**–**e** H1581 (2 × 10^6^ cells) transfected with LV-miR-214-3p or LV-NC in a volume of 50 μL (phosphate-buffered slaine (PBS):Matrigel = 4:1) were injected into the left lung of 6-week-old male BCLB/C nude mice (*n* = 20). One week after injection, five mice injected with LV-miR-214-3p and five mice injected with LV-NC were treated with FGFR1 inhibitor AZD4547 (12.5 mg/kg/day) randomly for 3 weeks. **c** Representative images showing tumor formation in the nude mice treated with LV-NC, AZD4547, LV-miR-214-3p, or LV-miR-214-3p combined with AZD4547 for 3 weeks. **d** Metastatic tumors in right lung and chest wall. **e** Survival curve for the mice in each treatment group evaluated. *P* values were calculated by Student’s *t* test: ***p < 0.001; ****p < 0.0001

Because the addition of the FGFR1 inhibitor AZD4547 actually led to downregulation of the tumor suppressor miR-214-3p in H1581 and DMS114 cells, we hypothesized that the downregulation of miR-214-3p partially accounted for the modest efficacy of AZD4547. To verify this hypothesis, H1581 cells were treated with increasing concentration of AZD4547, ranging from 0.01 to 10 μM, in combination with miR-214-3p or miR-NC using at a weak, fixed concentration (0.3 nM). The half-maximal inhibitory concentration (IC50) for AZD4547 plus miR-NC was 0.1995 μM. However, the IC50 for AZD4547 plus miR-214-3p was reduced to 0.01299 μM. To further explore the interaction of miR-214-3p and AZD4547, we calculated the combination index (CI) between them at different concentrations (CI = CA,*x*/IC*x*,A + CB,x/IC*x*,B). The range of CI values was below 0.2 (CI <1), indicating a strong synergy between miR-214-3p and AZD4547 (Fig. 7a). In the DMS114 cell line, the IC50 for AZD4547 plus miR-NC was 0.361 μM; however, the IC50 for AZD4547 plus miR-214-3p was decreased to 0.0245 μM. The range of CI values was below 0.4 (CI <1) (Fig. S4).

To further verify the synergism in vivo, subcutaneous mouse models and orthotopic lung cancer mouse models were established using H1581(Fig. 7b–e) and DMS114 cells (Fig. S4). After a week, miR-214-3p antagomir or miR antagomir NC was injected into the implanted tumor every 3 days for a total of seven injections in subcutaneous mouse models. Tumor volume was measured every 3 days. The results indicated that the subcutaneous tumors in miR-214-3p antagomir group grew slower than the miR-NC antagomir group. Meanwhile, the miR-214-3p antagomir plus AZD4547 group grew even slower than the miR-214-3p antagomir group or the AZD4547 group (*p* < 0.05, Fig. 7b).

The orthotopic lung cancer mouse models were established by ad-miR-214-3p virus-transfected cells. The volume of primary lung cancer and the number of metastatic nodules both decreased in an orderly fashion for the following four groups: the LV-miR-NC group, the AZD4547 group, the LV-miR-214-3p group, and the LV-miR-214-3p group plus AZD4547 group (*p* < 0.05, Fig. 7c, d). The LV-miR-214-3p group plus AZD4547 group had the longest survival time (Fig. 7e). Hematoxylin and eosin staining was used to quantify the number of nodules in the contralateral lung metastasis (Fig. S4). Immunofluorescence staining for the representative EMT markers were also applied. In the LV-miR-214-3p group plus AZD4547 group, FGFR1 and mesenchymal markers Snail and VIM were significantly suppressed, while the epithelial marker E-cad was highly expressed (Fig. S4).

Overall, we demonstrated the antitumor synergism between miR-214-3p and AZD4547 in vitro and in vivo.

## Discussion

The vital role of miRNAs in tumor initiation and progression has been highlighted in recent studies. MicroRNAs mainly inhibit the processes of post-transcriptional translation as well as the stability of mRNAs, controlling the cellular processes of inflammation, apoptosis, differentiation, migration, cell cycle regulation, and stress response by targeting related genes. We used a panel to identify miRNAs associated with tumor invasion and metastasis in lung cancer patients in this study. Ten miRNAs involved in tumor invasion and metastasis most frequently were detected in tumor tissues vs. NATs (*n* = 30)^36–40^. Among them, miR-214-3p expression was significantly lower in tumor tissues than NATs, which might act as a tumor biomarker. More importantly, miR-214-3p was correlated to a favorable patient prognosis, indicating that miR-214-3p may act as a biomarker to predict the recurrence, and outcome for lung cancer patients.

We subsequently performed a profiling study on the proliferation, invasion, migration, and EMT of FGFR1-amplified cell lines, which confirmed the tumor-suppressive effect of miR-214-3p. miR-214 was first reported to be developmentally related, which is subsequently shown to promote the progression and metastasis of melanoma^41,42^. However, previous studies have shown that miR-214-3p could be tumor promoter or suppressor in different tumor types. On the one hand, miR-214-3p increased the resistance to erlotinib and stimulated tumor proliferation and invasion by targeting LHX6 in patients with LADC^43^. Anti-miR-214 therapy has been shown to inhibit tumor progression in primary breast cancer tumors melanoma and pancreatic neuroendocrine cancer^44^. On the other hand, miR-214-3p is downregulated and suppresses tumor proliferation and metastasis in other tumors such as cutaneous squamous cell carcinoma and esophageal squamous cell carcinoma^45,46^. These reports indicate that the biological role of miR-214-3p varies up to the genetic background.

Recent studies indicated that miR-214-3p inhibited proliferation or tumor progression by targeting FGFR1 in the differentiation process of osteogenic mesenchymal stem cells and colorectal cancer, which was consistent with our research^47,48^. We confirmed that FGFR1 was a direct target of miR-214-3p and mediated the biological function of miR-214-3p in FGFR1-amplified lung cancer cells.

Wnt signaling mainly regulates development and stemness. The Wnt signaling is commonly divided into independent (non-canonical) signaling and β-catenin-dependent (canonical). Wnt signaling is associated with cancer stemness and metastasis. The role of Wnt signaling in carcinogenesis has most prominently been described for colorectal cancer, but aberrant Wnt signaling is observed in gastrointestinal cancers, leukemia, melanoma, breast cancer, and so on^49^. To further explore the functional pattern of miR-214-3p in FGFR1-amplified lung cancer, we performed GO data analysis to search the cross-signaling, and the Wnt signaling was found to be an intersection. Previous studies suggest that both miR-214-3p and FGFR1 could regulate MAPK/PI3K-AKT signaling^32^. Thus, we focused on Wnt/MAPK/PI3K-AKT signaling. We confirmed that miR-214-3p inhibited the activity of Wnt/MAPK/PI3K-AKT signaling, and overexpression of FGFR1 offset this effect, which suggest that miR-214-3p inhibited Wnt/MAPK/PI3K-AKT signaling pathway by suppression of FGFR1. Meanwhile, we also demonstrated that the Wnt signaling pathway was regulated by FGFR1. It was well known that Wnt/MAPK/PI3K-AKT signaling pathway promoted the progression of EMT in lung cancer, prostate cancer, nasopharyngeal carcinoma, cervical cancer, breast cancer, and so on^50–54^. Therefore, we, for the first time, proposed that existence of miR-214-3p-FGFR1-Wnt/MAPK/PI3K-AKT axis in FGFR1-amplified lung cancer.

It has long been recognized that FGFRs are overexpressed in many types of cancer cells, including NSCLC, breast cancer, and oral SQCC^35,55–57^. As mentioned above, FGFR1 as an attractive candidate target showed modest response. Therefore, genes’ co-alternation with FGFR1 or signaling paradigm have been widely studied, such as the co-active receptor tyrosine kinases, the co-activation of MTOR (mechanistic target of rapamycin) pathway, and so on.^58,59^ Our previous studies also confirmed that FGFR1 co-expressed with Gli2, SOX2, and YAP to maintain stemness or EMT in lung cancer^21,60^. Here, we confirmed the antitumor synergism between miR-214-3p and AZD4547 in vitro and in vivo. In the cell experiment, the CI index is about 0.2, showing a strong synergistic effect. Furthermore, subcutaneous and orthotopic lung cancer models also confirmed the synergistic effect.

The underlying molecular mechanism of the synergy between miR-214-3p and AZD4547 has remained unknown. Interestingly, we found a loop in which FGFR1 can simultaneously regulate the level of miR-214-3p through ERK. FGFR1 inhibitor AZD4547 reduced the level of miR-214-3p by suppressing ERK activation in cell lines. However, it was confirmed that miR-214-3p acted as a tumor suppressor by targeting FGFR1. Therefore, FGFR1 inhibitor AZD4547 might upregulate the level of FGFR1 and the pathway downstream of FGFR1 by inhibiting miR-214-3p. Meanwhile, we did notice that a dynamic balance between miR-214-3p and FGFR1 existed within 96 h after the addition of AZD4547, which need further exploration. Previous studies identified DUSP6 deletion and NRAS amplification in drug-resistant H1581 cells, leading to the reactivation of MAPK pathway. Meanwhile, drug-resistant DMS114 cells drove MAPK pathway reactivation by suppressing upregulation of MET^61^. Therefore, in future studies it is critical to investigate the relationship between DUSP6, NRAS, miR-214-3p, and MAPK signaling in drug-resistant H1581 cells, and the correlation of MET, miR-214-3p, and MAPK signaling in drug-resistant DMS114 cells.

The microRNA mimic has been discovered and employed for cancer therapy in the clinical setting since 2013. In addition, clinical trials of microRNAs for patient prognosis and treatment are currently underway^62^. As stated above, our findings in this study have demonstrated that miR-214-3p acts as a vital biological targeting inhibitor for FGFR1, which need further study for cancer treatment.

In summary, in this study we have discovered a negative feedback regulatory axis between miR-214-3p and FGFR1, as well as a synergistic antitumor effect between miR-214-3p and AZD4547. These findings may provide new insights for the prognosis and treatment of patients with FGFR1-amplified lung cancer.

## Materials and methods

### Cells and reagents

H1581, DMS114, and HCC95 were purchased from the American Type Culture Collection and were authenticated by STR profiling (Table. S1)^21^. The cell lines were cultured with RPMI-1640 (HyClone) with 10% fetal bovine serum (FBS) (Gibco). FGFR1 inhibitor AZD4547 was kindly provided by AstraZeneca Pharmaceutical Company^63^. AZD6244 was obtained from Selleck Chemicals^64^. FGF2 was purchased from PeproTech.

### miRNA, siRNA transfection, and plasmid construction

The miRNA mimics, inhibitors, and antagomir were obtained from RiboBio (Guangzhou, China). miRNA NC, miRNA mimics, and miRNA inhibitors were transfected by Lipofectamine 3000 (Invitrogen, Carlsbad, USA). The ad-miR-214-3p virus was purchased from Hanbio Biotechnology Co. Ltd. (Shanghai, China). The FGFR1 overexpression plasmid was purchased from biolink (Shanghai, China). The sequences of siFGFR1, siRNA-NC, WT ERK2, and autophosphorylation variant ERK2 p.R67S (ERK2_R67S) were described by us before^21^.

### Colony formation assay

Cells were transfected into miR-214-3p mimics and inhibitors. After 24 h, cells were digested with trypsin and replanted at a density of 1000 cells/10 cm^2^. After 3 weeks of incubation, cells were fixed with methanol and then stained with 0.1% crystal violet. Colonies larger than 100 µm in diameter were counted, and each assay was repeated in triple.

### Quantitative real-time PCR

Lung cancer tissues NATs) were provided by Shanghai Chest Hospital, Jiao Tong University (Shanghai, China). Written, informed consents approving the use of tissue samples for research purposes were obtained from lung cancer patients. The study was approved by the Institute Research Ethics Committee of Shanghai Jiao Tong University^60^. Total RNA was isolated from the above cells or frozen tissues using Trizol reagent (Invitrogen). Complementary DNA was prepared with 2 ng of total RNA for miRNA and mRNA quantification using a Mir-X miRNA First-Strand Synthesis Kit (Clontech Laboratories Inc., USA) and PrimeScript^TM^ RT Master Mix Kit (Takara, Dalian, China). RNA expression was measured by qRT-PCR using a Mir-X miRNA qRT-PCR SYBR Kit (Clontech Laboratories Inc., USA) and a SYBR^®^ Green (Takara, Dalian, China), according to the manufacturer’s protocol. U6 or GAPDH (glyceraldehyde 3-phosphate dehydrogenase) was used for the normalization. The primers for miRNA and U6 were purchased from RiboBio Company (Guangzhou, China). The sequences of the primers are covered by a patent. The primer sequences of this study were as follows:

ZO-1 (forward, 5′-AGCGAAGCCACCTGAAGATA-3′ and reverse, 5′-GATGGCCAGCAGGAATATGT-3′).

Snail (forward, 5′-CGCGCTCTTTCCTCGTCAG-3′ and reverse, 5′-TCCCAGATGAGCATTGGCAG-3′), the other primer sequences were described by us before^21^.

### Scratch assay and Transwell migration/invasion assay

Scratch assay was described by us before^21^. Cells (5 × 10^4^) with 1% FBS medium were seeded in an 8-μm pore membrane with Matrigel-coated or not (Corning, 356231), and 10% FBS medium was placed in the lower chamber. After 36 h incubation, cells on the upper layer were removed using a cotton swab. Passing through cells were fixed in methanol for 20 min and then stained for 20 min with 0.1% crystal violet dye. The stained cells were counted at three randomly selected views for subsequent calculations.

### CCK8 assay

CCK-8 assay kit (Dojindo Laboratories, Kumamoto, Japan) was used to examine the proliferation rate under treatment. Briefly, 2 × 10^3^ cells were seeded into 96-well plates. After transfected with miR-214-3p mimic or miR-214-3p inhibitor for 24 h, 10 μL CCK8 reagent was added into the medium per well. The absorbance at 450 nm was measured by the microplate reader (Synergy2, BioTek, Winooski, VT) after 1 to 3 h coincubation.

### Western blot

Treated cells or tissues pulverized by ultrasonic waves were lysed by RIPA containing the Complete Protease Inhibitor Cocktail. Phosphatase Inhibitor Cocktail and phenylmethylsulfonyl fluoride. After centrifugation at 12,000 r.p.m. for 20 min at 4 °C, the supernatant was procured for the next step. An equal amount of 30 μg protein was separated by 10% sodium dodecyl sulfate-polyacrylamide gel electrophoresis gel and then transferred onto NC membranes (Millipore, Billerica, MA, USA). After blocking with 5% bovine serum albumin for 1 h, antibodies were incubated overnight at 4 °C. The intensity of the signal was detected by the ECL chemical imager^21^. All the antibodies were purchased from Cell Signaling Technology (Beverly, MA, USA).

### Immunofluorescence microscopy

The tissue sections were incubated with primary antibody overnight after being rehydrated. The secondary antibody was donkey anti-rabbit immunoglobulin G conjugated with Alexa Fluor 594. Cell nuclei were stained with 4′,6-diamidino-2-phenylindole (Sigma) Stained cells were photographed and quantified under an immunofluorescence microscope (Leica DFC420C).

### Immunohistochemistry

After rehydrating, tissue specimens were incubated with primary antibodies for 1 h, followed by incubation of the secondary antibodies. Diaminobenzidine-hydrogen peroxide (Sigma) was the chromogen, and the counterstaining was carried out with 0.5% hematoxylin. The degree of IHC was classified into the following grades (−, no staining; +, 10%; ++, 10–50%; and +++, >50%)

### In vivo xenograft assay and orthotropic lung tumor model

In the subcutaneous xenograft model, H1581 (1 × 10^6^ cells) or DMS114 (2 × 10^6^ cells) were injected into the right side of the nude mouse in a volume of 50 µL. AZD4547 (12.5 mg/kg/day) was given to nude mice by gavage for 3 weeks. In the orthotopic xenograft model, H1581 (2 × 10^6^ cells) or DMS114 (3 × 10^6^ cells) cell suspensions in a volume of 50 μL were injected through the intercostal space into the left lung of nude mice. AZD4547 (12.5 mg/kg/day) was given by gavage for 2 weeks. All the nude mice were housed in the SPF (specific pathogen free) animal room of Shanghai Jiao Tong University. All animal experiments were carried out in accordance with the approved scheme of the Shanghai Jiao Tong University Institutional Ethics Committee.

### Luciferase reporter assays and transient transfection

The 3′-UTR sequence of FGFR1 interacting with miR-214 and the corresponding mutant sequence within the predicted target site were predicted to be synthesized and inserted into the pmiR-GLO dual-luciferase miRNA target expression vector (Guangdong Ruibo Biotech Co. Ltd.) called WT-FGFR1 3′-UTR and mt-FGFR1 3′- UTR. Subsequently, H1581 or DMS114 cells were plated into 24-well plates and transfected with these vectors. Cells were co-transfected with miR-214 and WT-FGFR1 3′-UTR, miR-214 and MT-FGFR1 3′-UTR, miR-NC and WT-FGFR1 3′-UTR, and miR-NC and mt-FGFR1 3′-UTR. After 48 h, cells were measured according to the procedure of manufacturer’s instructions (Dual-Luciferase Assay System; Promega). pRL-TK expressing Renilla luciferase is co-transfected to use as an internal reference.

### Analysis of public datasets from TCGA

Prognostic values of miR-214-3p level were downloaded from the TCGA database and analyzed by Kaplan–Meier survival curves of NSCLC patients, using Kaplan–Meier plotter (www.kmplot.com/analysis).

### Statistical analysis

All the data were presented as mean ± SEM or SD and analyzed using the GraphPad Prism software (version7.0a, GraphPad Software Inc.). All experiments have three independent operations. We used Student’s *t* tests to compare changes in miRNA levels between tumor tissues and corresponding paired normal tissues. *P* value <0.05 was considered statistically significant.

## Supplementary information

SUPPLEMENTAL TABLE 1

SUPPLEMENTARY FIGURE LEGENDS.

Supplementary Figure 1.

Supplementary Figure 2.

Supplementary Figure 3.

Supplementary Figure 4.
