# Supplementary material for: Reciprocal regulatory mechanism between miR-214-3p and FGFR1 in FGFR1-amplified lung cancer
Source: Oncogenesis. 2019 Sep 6;8(9):50. doi: 10.1038/s41389-019-0151-1 (PMC6731303; doi:10.1038/s41389-019-0151-1)
Supplement: Supplementary file 2 — SUPPLEMENTARY FIGURE LEGENDS. [file 41389_2019_151_MOESM2_ESM.docx]

**SUPPLEMENTARY FIGURE LEGENDS**

**Supplementary figure1**

(a)10 miRNA levels was detected by qRT-PCR in lung cancer patients(n=30).（b）The expression level of FGFR1 was detected by western blot. *P values were caculated by Student t-test. * p < 0.05; ** p < 0.01; *** p < 0.001; **** p < 0.0001.*

**Supplementary figure2**

H1581 , DMS114 and HCC95 cell lines were transfected with miR-214-3p inhibitor or Anti-miR-NC. (a,b) Cell growth was measured by the CCK8 assay and clony assay. (c,d) Migration and invasion was determined by scratch assay and transwell assay. (e) Quantification of Represent EMT markers was measured by western blot. P values were caculated by Student t-test. * p < 0.05; ** p < 0.01; *** p < 0.001; **** p < 0.0001.

**Supplementary figure3**

（a,b）The hallmarks and signatures in the groups with 25% high and low expression of FGFR1 and miR-214-3p in the TCGA lung cancer cohort，which were determined by a normalized enrichment score (NES);(c,d)The expression level of miR-214-3p was detected by qRT-PCR after treatment with (c)Wnt signaling inhibitor XAV-939 or (d)AKT inhibitor MK-2206 2HCl *P values were caculated by Student t-test. * p < 0.05; ** p < 0.01; *** p < 0.001; **** p < 0.0001.*

**Supplementary figure4**

(a)DMS114 cells were transfected with 0.3 nM miR-NC or miR-214-3p, incubating with AZD4547 in a serial dilution. After 72 hours, CCK8 was used to evaluate cellular proliferation. CI values were calculated (b) Representative images showing tumor formation in the nude mices treated with miRNA agomir NC、AZD4547、miR-214-3p agomir or miR-214-3p and agomir for 3 weeks(combine).(c,d,e,f) The orthotopic lung cancer mouse models were established (c) Representative images showing tumor formation in the nude mice treated with LV-NC、AZD4547、LV-miR-214-3p or LV-miR-214-3p combined with AZD4547 for 3 weeks(d) Quantification of Represent EMT markers was measured by Immunofluorescence. The metastasis tumor nodes in lungs were presented by HE staining.（e）Metastatic tumors in right lung and chest wall.（f）Survival curve for the mice in each treatment group evaluated. *P* values were caculated by Student t-test. * *p* < 0.05; ** *p* < 0.01; *** *p* < 0.001; **** *p* < 0.0001.
