# Supplementary figures and images for: Reciprocal regulatory mechanism between miR-214-3p and FGFR1 in FGFR1-amplified lung cancer
Source: Oncogenesis. 2019 Sep 6;8(9):50. doi: 10.1038/s41389-019-0151-1 (PMC6731303; doi:10.1038/s41389-019-0151-1)

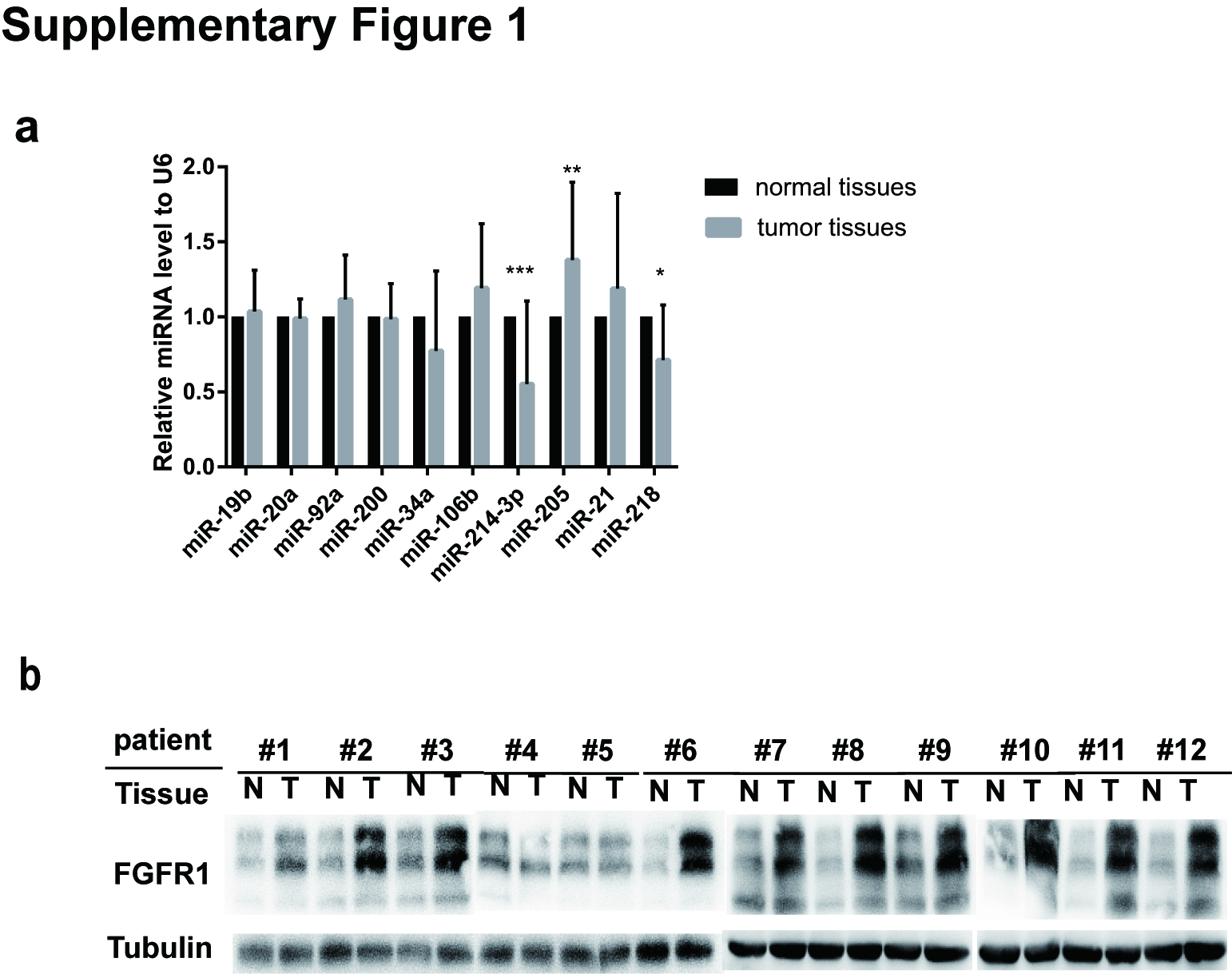

Supplement: Supplementary file 3 — Supplementary Figure 1. [file 41389_2019_151_MOESM3_ESM.tif]

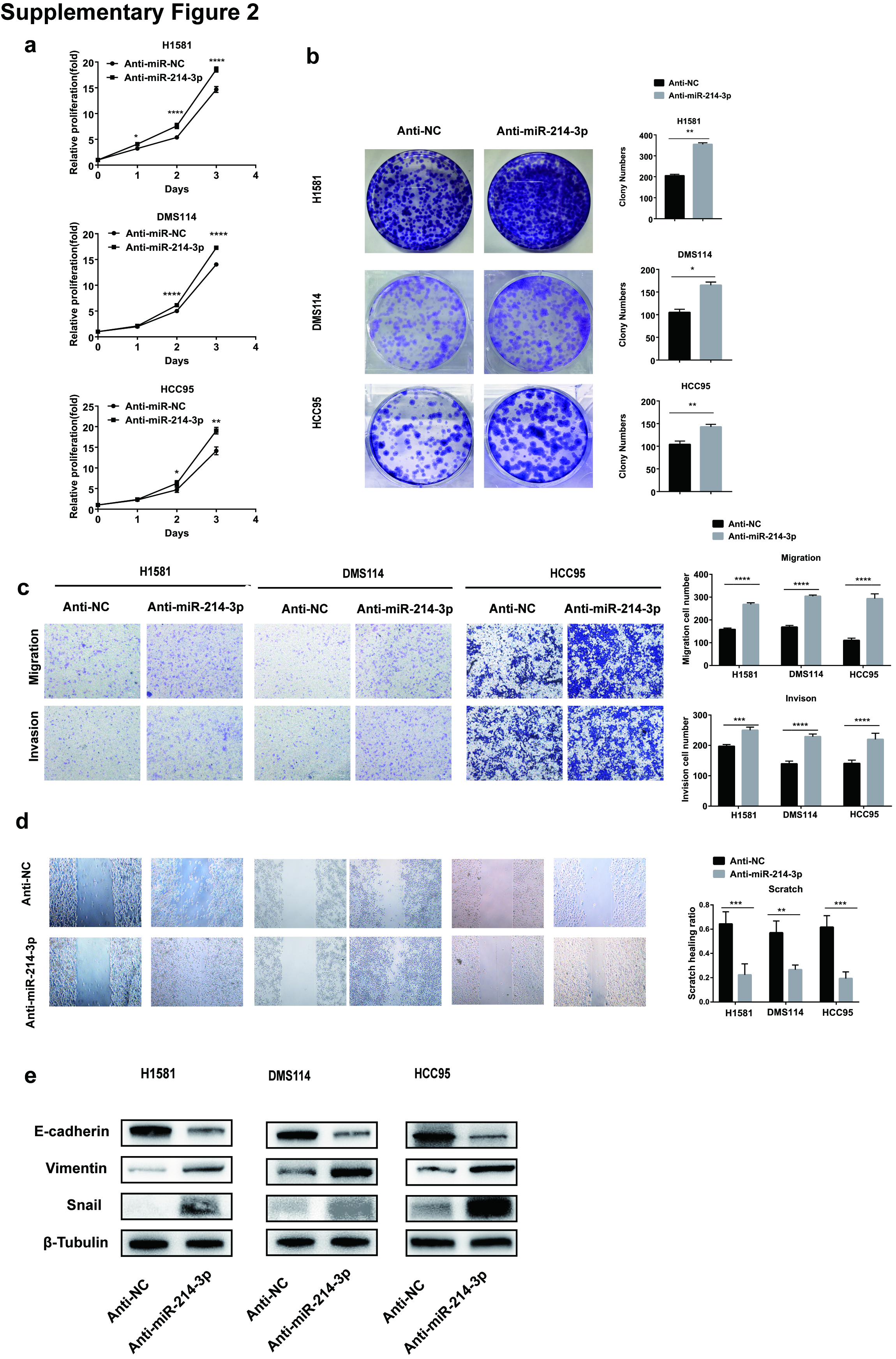

Supplement: Supplementary file 4 — Supplementary Figure 2. [file 41389_2019_151_MOESM4_ESM.tif]

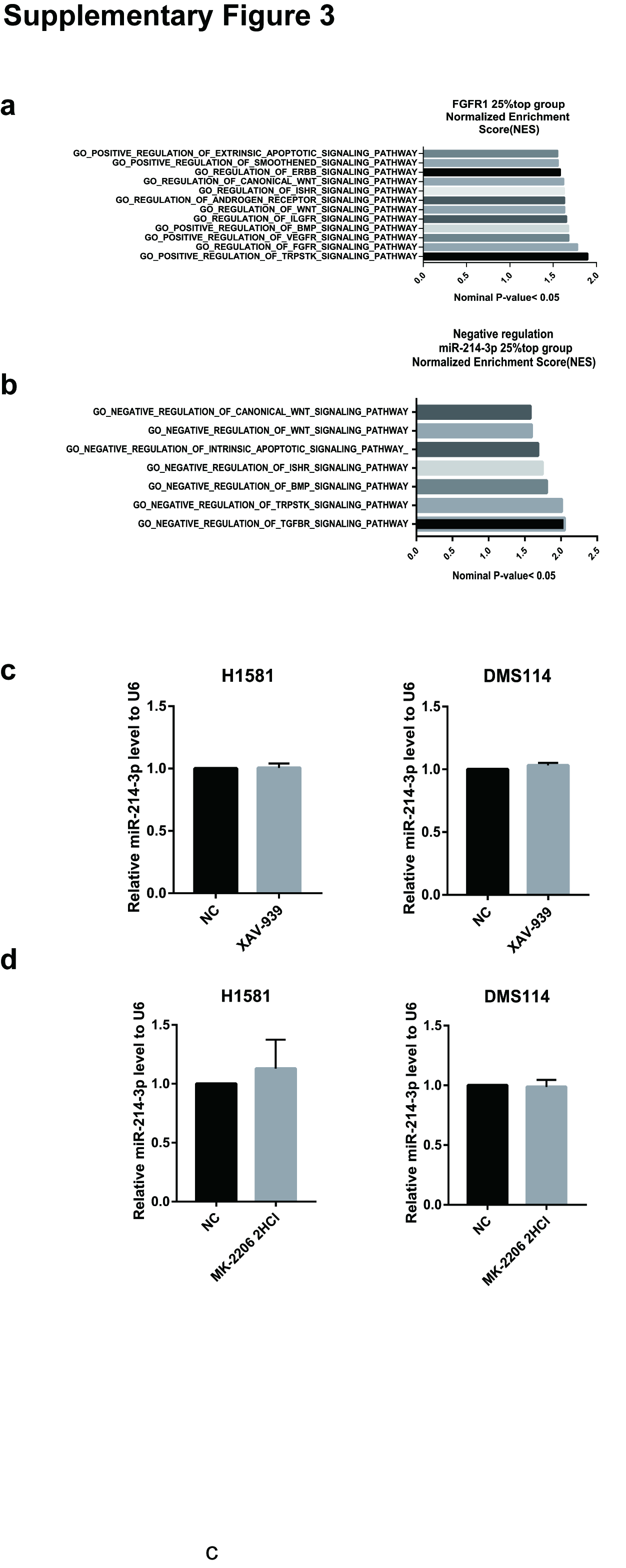

Supplement: Supplementary file 5 — Supplementary Figure 3. [file 41389_2019_151_MOESM5_ESM.tif]

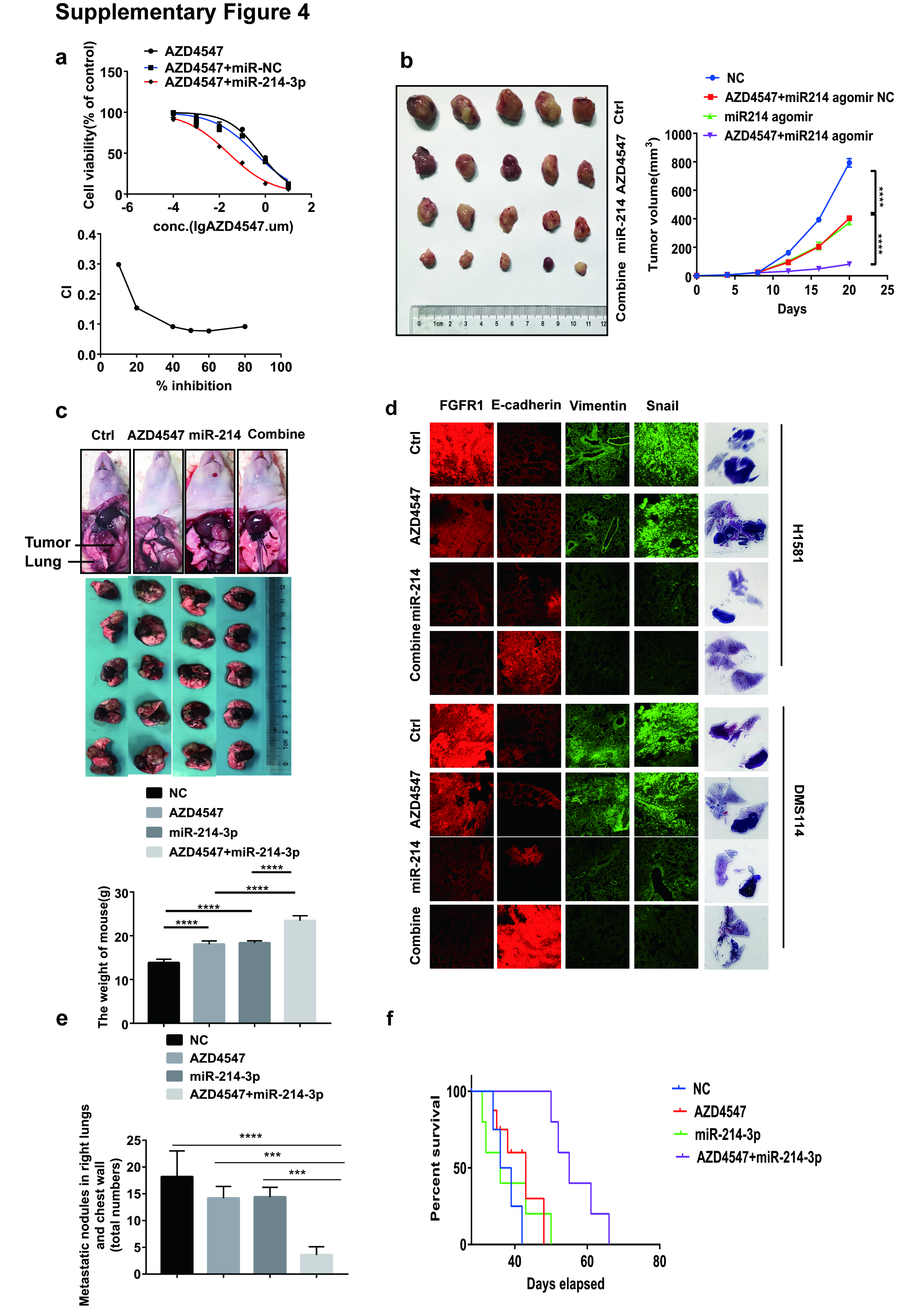

Supplement: Supplementary file 6 — Supplementary Figure 4. [file 41389_2019_151_MOESM6_ESM.tif]
